# Dinosaur senescence: a hadrosauroid with age-related diseases brings a new perspective of “old” dinosaurs

**DOI:** 10.1038/s41598-021-91366-1

**Published:** 2021-06-11

**Authors:** Justyna Słowiak, Tomasz Szczygielski, Bruce M. Rothschild, Dawid Surmik

**Affiliations:** 1 Institute of Paleobiology, Polish Academy of Sciences, Twarda 51/55, 00-818 Warsaw, Poland; 2 Carnegie Museum of Natural History, 4400 Forbes Ave, Pittsburgh, PA 15213 USA; 3 Institute of Earth Sciences, Faculty of Natural Sciences, University of Silesia, Będzińska 60, 41-200 Sosnowiec, Poland

**Keywords:** Animal physiology, Palaeontology

## Abstract

Senile vertebrates are extremely rare in the fossil record, making their recognition difficult. Here we present the largest known representative of the Late Cretaceous hadrosauriform *Gobihadros mongoliensis* showing features of cessation of growth indicating attainment of the terminal size. Moreover, this is the first non-avian dinosaur with an age-related pathology recognized as primary calcium pyrophosphate deposition disease indicating its advanced age. Because senile dinosaurs are so rare and thus “senescence” in dinosaurs is unclear, we also propose a new unified definition of a senile dinosaur: an individual which achieved the terminal size as revealed by the presence of the external fundamental system and closed transcortical channels, has completely secondary remodeled weight-bearing bones and possesses non-traumatic, non-contagious bone pathologies correlated with advanced age.

## Introduction

Assessing ontogenetic age in non-avian Dinosauria is complicated^1,2^. Overall, three ontogenetic stages are commonly distinguished: juvenile, subadult, and adult. The features distinguishing them are connected to body size, advancement of skeletal ossification, and bone microstructure. In general, juvenile individuals show features indicating lack of somatic maturity, e.g., incipient skeletal fusion and bone tissues indicative of fast growth rate. Subadults show a mixture of juvenile and adult features. Adults show severe slowdown or stoppage of growth (expressed by the external fundamental system, EFS, i.e., a set of closely positioned lines of arrested growth in the external-most cortex^3^ or the outer circumferential layer, OCL, i.e., avascular bone composed of slowly deposited parallel-fibered bone^4^). Other adult features include fusion of skeletal elements (particularly cranium and vertebrae), proportionally smaller orbits, larger number of teeth, large body size, medullary bone (bone tissue around the medullary cavity, being a calcium reservoir for building the hard eggshell in females before laying eggs)^5^, and well developed sociosexual features^1,2^. However, non-avian dinosaurs tend to show a mosaic of features hampering their age estimation, so the classification of ontogenetic stages depends on authors’ interpretation^2^. In addition, numerous other ontogenetic classes were distinguished to date in the literature, such as embryo, perinate, small and large nestling, young, fully grown, old adult, and senile^1,2^. The latter three were used interchangeably referring to exceptionally large individuals, but their definitions are ambiguous. In non-avian dinosaurs these classes were usually suggested based on large size^6^; nearly all cranial sutures ossified^7^; fusion of axial elements^6,8^; ossification of the postcranial bones^6^; presence of the EFS^9–11^; presence of the outer circumferential layer^12^; and/or multiple generations of secondary osteons^13^. Given natural size variability, large size alone does not necessarily indicate advanced ontogenetic age, without combination with other features^2^. Thus, identification of a senile dinosaur is difficult, especially that exceptionally large and/or ontogenetically old individuals are rare in the fossil record^9,14^, which may be connected to predator pressure, taphonomy, diseases, and higher mortality of immature individuals^14,15^. Based on comparisons between the lifespans of wild and captive animals, it can be inferred than most species have a significantly shorter life expectancy in the natural environment, thus rarely attaining senescence^16^. Since the specimen of *Gobihadros mongoliensis* described herein shows features indicating its advanced age in combination with age dependent pathology, it gives a unique insight into the ageing process of non-avian dinosaurs.

## Results

### Institutional abbreviations

ZPAL, Institute of Paleobiology, Polish Academy of Sciences, Warsaw, Poland. MPC, Mongolian Paleontological Center, Ulaanbaatar, Mongolia.

### Systematic paleontology

Ornithischia Seeley, 1887.

Ornithopoda Marsh, 1881.

Iguanodontia sensu Sereno, 1998.

Hadrosauroidea sensu Sereno, 1998.

*Gobihadros mongoliensis* Tsogtbaatar et al., 2019.

### Referred material

Fragmentary femur, tibia, pedal phalanges, nine caudal vertebrae, and other fragmentary bones (ZPAL MgD-III/3).

### Geological setting

The Baynshire Formation estimated to be late Cenomanian to Santonian in age; Khongil Tsav locality, Mongolia.

### Description

**Figure 1:**
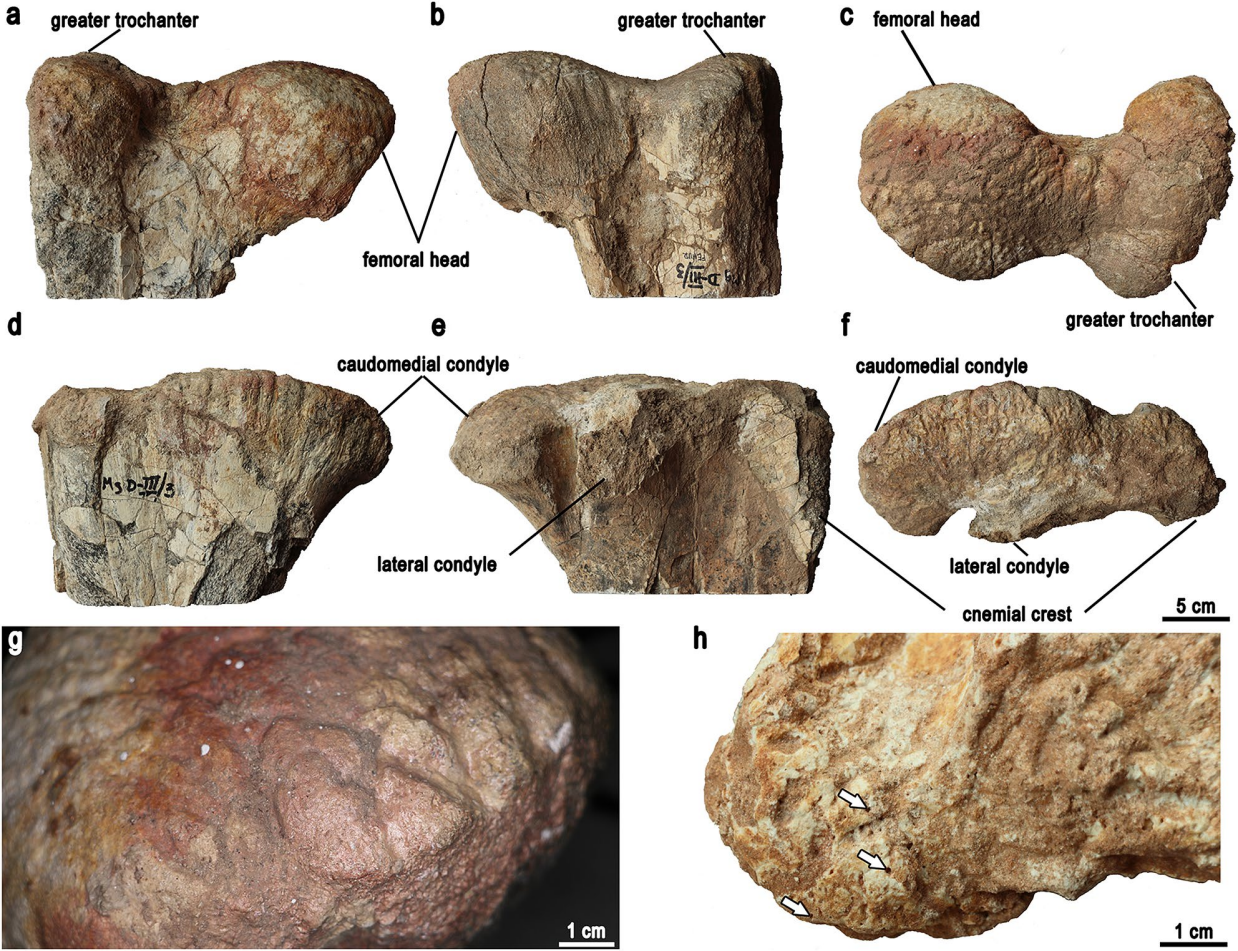
Proximal right femur (**a**–**c**) and tibia (**d**–**f**) of *Gobihadros mongoliensis*. Proximal right femur in cranial (**a**), caudal (**b**), and dorsal (**c**) view. Proximal right tibia in medial (**d**), lateral (**e**), and dorsal (**f**) view. Surface of the proximal femur without transcortical channels (**g**). Left distal femur of *Protoceratops andrewsi* (ZPAL MgD-II/11) showing open transcortical channels marked by arrows (**h**).

The proximal and distal parts of the right femur (Fig. 1a–c) and the proximal end of the right tibia (Fig. 1d–f) are preserved. The bones fit the shape of other specimens of *Gobihadros mongoliensis*^17^. Transcort ical channels (vascular channels perforating the cortices of the articular surfaces and nourishing the epiphyseal plates responsible for bone elongation; Fig. 1h) are not detected on the articular surfaces of both bones (Fig. 1g). See the Supplementary Materials for detailed description.

**Figure 2:**
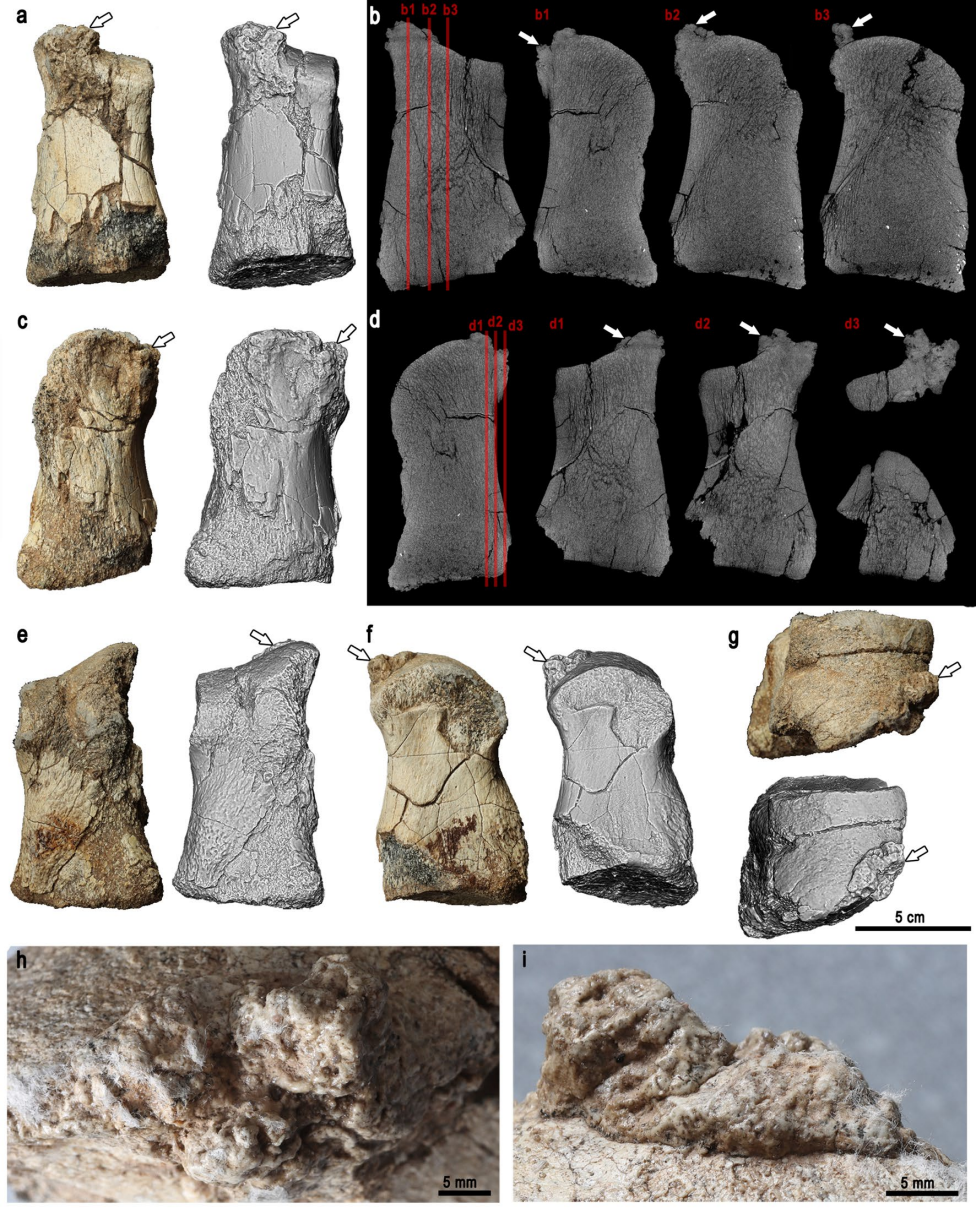
Phalanx 1 of digit IV. (**a**) Dorsal view. (**b**) Dorsoventral CT sections, planes of sectioning indicated by red lines on the lateromedial CT section. (**c**) Lateral view. (**d**) Lateromedial CT sections, planes of sectioning indicated by red lines on the dorsoventral CT section. (**e**) Ventral view. (**f**) Medial view. (**g**) Distal view. White arrows indicate the calcium deposit. (**h**–**i**) Closeups of the calcium deposit.

Left foot elements are preserved. Only the medial half of phalanx 1 of the left digit II is preserved. The nearly symmetric phalanx 1 of left digit III is the largest. Phalanx 1 of the left digit IV is asymmetric; the lateral margin is longer than the medial (Fig. 2a–g). The proximal articular surface is flat and roughly trapezoid to subtriangular, with an especially pronounced medial margin. The dorsal surface of the phalanx is more concave than the lateral and ventral, while the medial surface is flat. The distal articular surface is slightly saddle-shaped, convex in mediolateral aspect. Its medial and ventral margins are straight, while the dorsolateral margin is continuous and gently bowed. The lateroventral part of the distal articular surface is not preserved. There is a surface calcific deposit on the middle aspect of the dorsolateral edge of the distal articular surface (Fig. 2h–i), diagnostic of calcium pyrophosphate deposition disease (CPPD)^18^. The general shapes of the first phalanges of the digits II, III, and IV fall into the spectrum of shapes known in other Hadrosauroidea^19^.

**Figure 3:**
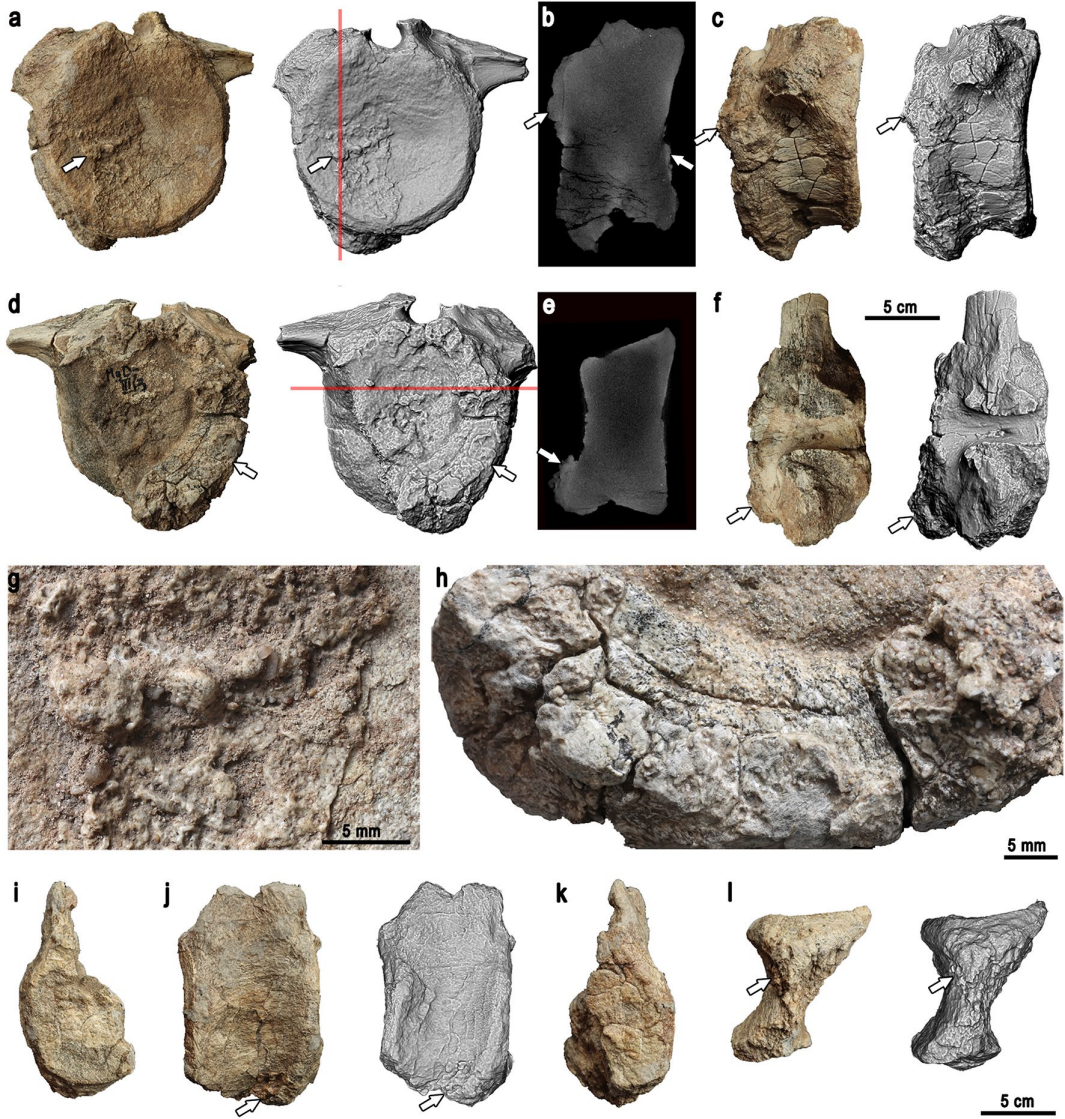
Pathological caudal vertebrae. (**a**–**h**) Proximal caudal vertebra. (**a**) Cranial view, red line indicating the plane of the CT section. (**b**) Sagittal CT section. (**c**) Right lateral view. (**d**) Caudal view, red line indicating the plane of the CT section. (**e**) Coronal CT section. (**f**) Dorsal view. (**g**–**h**) Closeups of the calcium deposit on the cranial (**g**) and caudal (**h**) surface. (**i**–**l**) Middle caudal vertebra. (**i**) Cranial view. (**j**) Right lateral view. (**k**) Caudal view. (**l**) Ventral view. White arrows indicate calcium deposit.

Parts of nine caudal vertebrae are preserved. Three of them represent the proximal part of the tail; four, middle; and the remaining two, distal. The proximal caudal centra are short craniocaudally, wide mediolaterally with larger distal than proximal surfaces for chevron articulation (Fig. 3a–f). The centrum of the proximal-most preserved vertebra is affected by calcium deposit, indicating CPPD^18^. The middle caudal centra have dorsoventral height similar to the craniocaudal length, prominent proximal and distal articular surfaces for the chevron and lack transverse processes. The chevron facet of one of the vertebrae is covered by a calcium deposit, indicating CPPD^18^ (Fig. 3i–l). Finally, the distal caudal vertebrae have cylindrical centra twice as long craniocaudally as high dorsoventrally. The morphology of all the vertebrae is the same as in the caudal vertebrae of the *Gobihadros mongoliensis* individuals already described^17^.

### The pathology

Pathology can be observed on the pedal phalanx 1 of digit IV (Fig. 2) and the proximal-most preserved caudal vertebra (Fig. 3). The affected area is restricted to the craniolaterodistal margin of phalanx 1 of digit IV. In distal view, there are calcific deposits on the otherwise smooth articular surface of the phalanx. The extent of the pathology is best visible in dorsal view (Fig. 2a). The lateral surface of the phalanx is not affected; only the distalodorsal margin bears signs of the lesions.

The surface phenomenon is prominent in the proximal-most preserved caudal vertebra, especially along the ventrocaudal margin of the centrum, less pronounced on the cranial surface of the same centrum (Fig. 3a–h). The left laterocaudal part of the centrum is missing, but calcium deposits are preserved along the whole caudal right edge. The calcium deposits expand onto the lateral right surface of the centrum. The expansion is greater in the dorsolateral part than in the ventrolateral area. The caudal surface of the centrum is damaged, so it is unknown how much of it was affected by the pathology. Cranially, the right side of the centrum bears a relatively thin layer of calcium deposits on its surface. The cranial left margin of the centrum does not show any pathological alterations. In CT images, the pathological areas have radiodensity comparable with that of unaffected cortex and appear compact (although clarity of the internal microstructure of the scanned vertebra is not optimal, most likely due to the high radiodensity of the mineralization within the intertrabecular spaces, similar to that of the bone; Fig. 3b,e). As in the case of the phalanx, separation of the calcium deposits from the main bulk of the centrum by a radiolucent line is visible throughout most of the scan. Calcium deposits cover the chevron facet of the middle caudal vertebrae and the ventral side of the vertebra close to the chevron facet. The caudal surface of the centrum is worn, so the presence of calcium deposits is unknown. However, the preserved fragment of the cranial surface of the vertebra shows calcium deposits (Fig. 3l).

**Figure 4:**
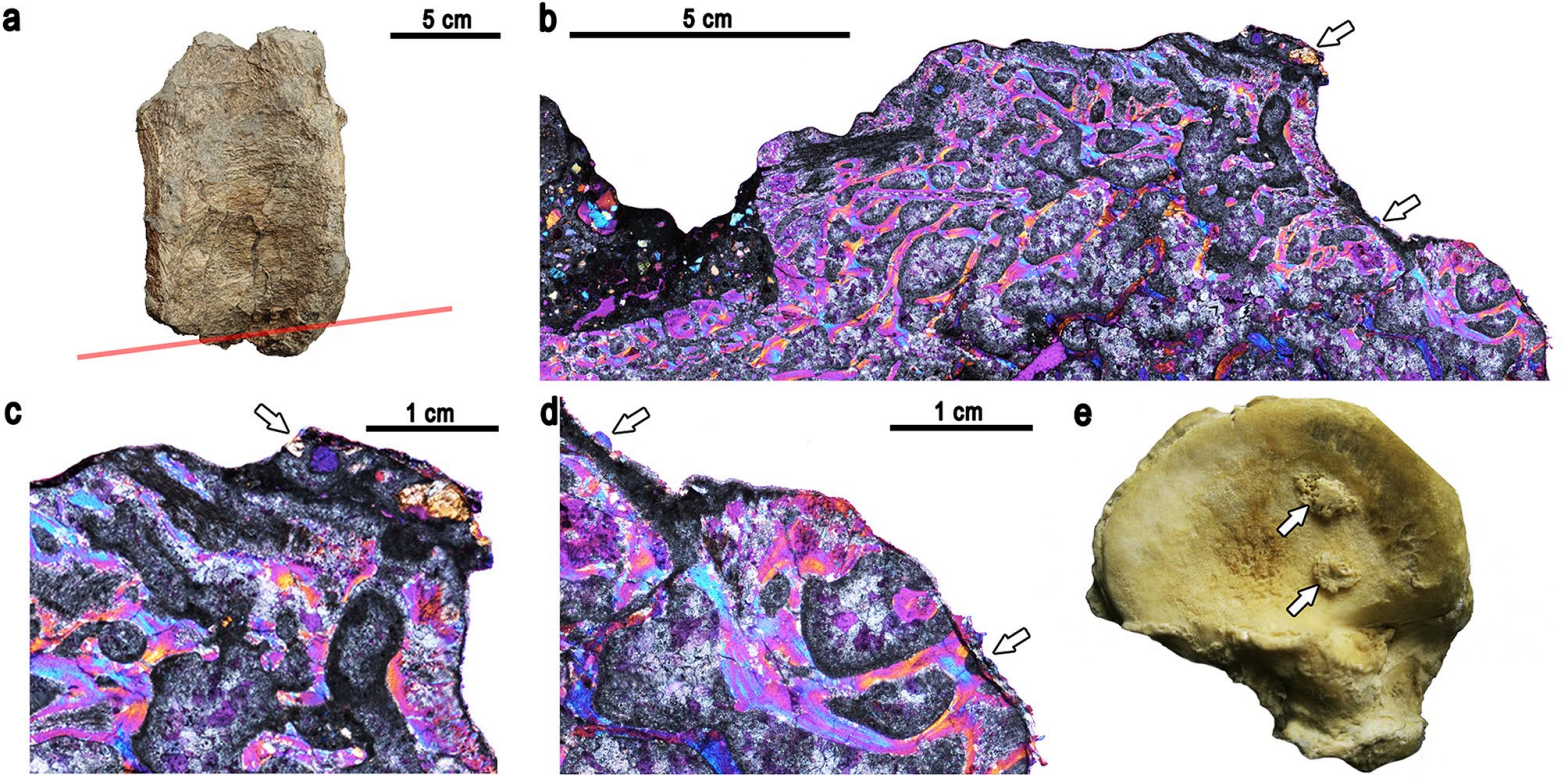
Calcium pyrophosphate deposition. (**a**) Pathological middle caudal vertebra with marked sectioned place. (**b**) The thin section under polarized light with gypsum wedge. (**c**, **d**) Close up of the pleiomorphic crystals under polarized light with gypsum wedge. (**e**) en face view of glenoid surface of scapula from # 2643 from Hamann-Todd human skeletal collection (Cleveland Museum of Natural History, Cleveland, Ohio, USA). Arrows are pointing at the calcium pyrophosphate crystals.

The middle-sized caudal vertebra was thin sectioned across the affected area (Fig. 4a). Overall, the sectioned bone microstructure is poorly preserved, making observation of microstructural characteristics difficult. The structure of the vertebral centrum is dominated by spongy bone composed of bony trabeculae, which are severely crushed due to diagenesis. The CPPD is underlaid by cancellous bone with variably sized openings. The calcium deposits are weakly developed in the sectioned specimen; nonetheless, pleomorphic crystals with blunt ends, characteristic for CPPD^20^, are present on the bone surface and best visible under polarized light with gypsum wedge (Fig. 4b–d). The blunt edges of the crystals clearly differentiate them form the crystals of the neighboring sediment (Fig. 4b).

## Discussion

**Figure 5:**
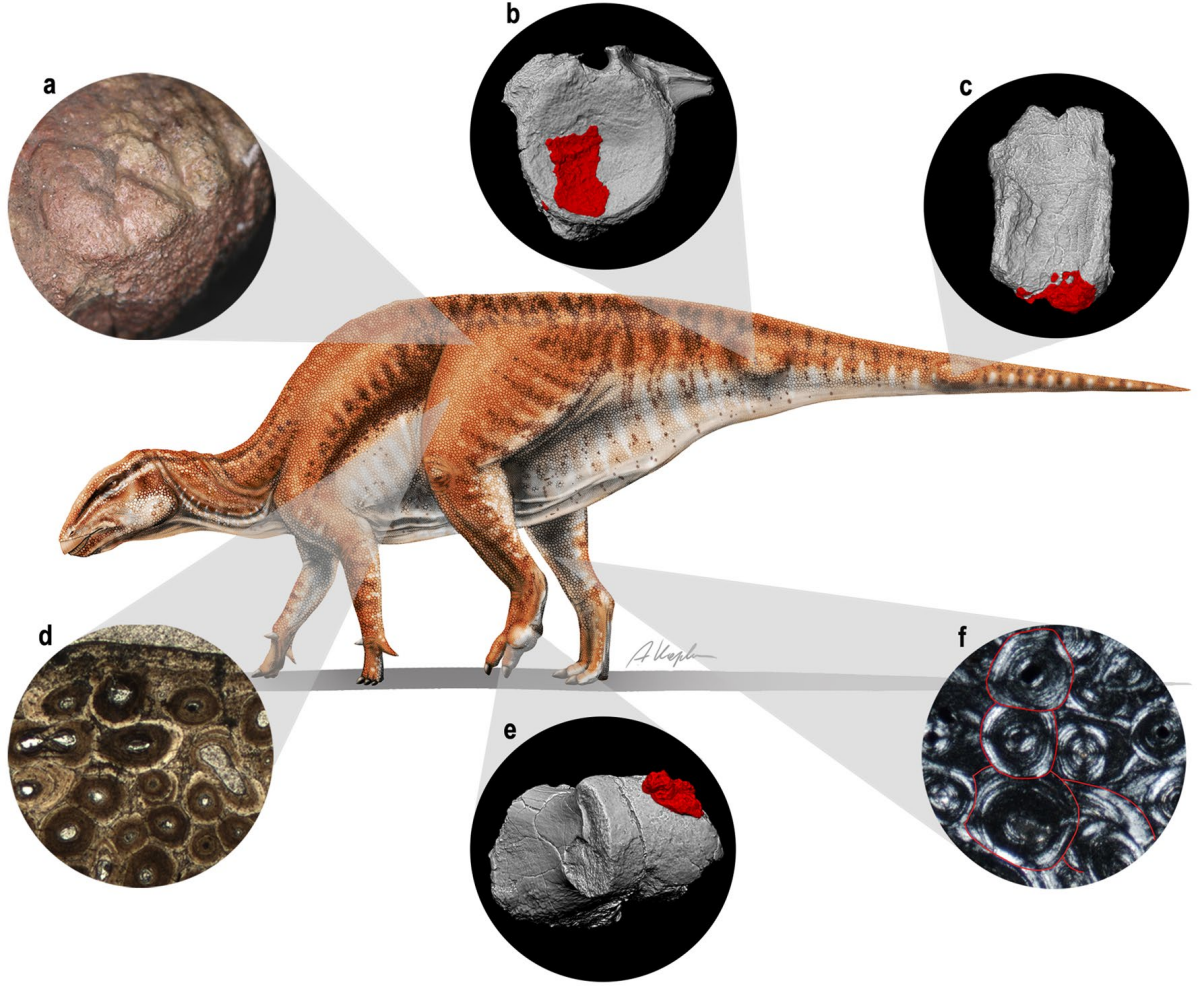
Life reconstruction of *Gobihadros mongoliensis* ZPAL MgD-III/3 with highlighted features indicating its senescent age. (**a**) Closed transcortical channels on th e proximal articular surface of the femur. (**b**) Calcium deposits on the distal surface of the proximal caudal vertebra. (**c**) Calcium deposits on the ventral surface of the middle caudal vertebra. (**d**) The presence of the EFS and extensive secondary remodeling in the external cortex of the femur. (**e**) Calcium deposits on the distal articular surface of the first phalanx of digit IV. (**f**) Four generations of secondary osteons in the middle cortex of the tibia. The reconstruction of *G. mongoliensis* drawn by A. Kapuścińska.

The diagnostic characters of *Gobihadros mongoliensis* provided by Tsogtbaatar et al.^17^ are restricted to the premaxilla, ilium, and digit I of the manus, which are not preserved in ZPAL MgD-III/3. *G. mongoliensis* is the only large ornithopod known from the Baynshire Formation in Khongil Tsav locality^17^. The morphologies of the preserved proximal femur, proximal tibia, and caudal vertebra of ZPAL MgD-III/3 fit that of numerous individuals of *G. mongoliensis* already known^17,21^. Thus, its identification as *G. mongoliensis* is the most plausible. Measurements of the proximal phalanges of the pes, proximal tibia, and proximal femur indicate that the individual was larger than the *G. mongoliensis* individuals reported so far^17^ (Table 1). The histological sampling of the femur of ZPAL MgD-III/3 revealed the presence of closely arranged lines of arrested growth in the external cortex (external fundamental system, Fig. 4d). Both the femur and the tibia present extensively secondarily remodeled cortex^21^ (Fig. 5f). The samples can be staged histologically as Histologic Ontogenetic Stage 14 and Remodeling Stage 11^13,22,23^. Note that the stages were designed for sauropods, not ornithopods, although remodeling in the middle and, particularly, deep cortex makes counting of the osteon generations difficult, possibly leading to underestimation. This is highlighted by some occurrences of newer osteons developing centered on the older, implying that at least in some cases the older generation are overwritten. Those features indicate advanced ontogenetic age of the individual^21^. That is further supported by the presence of closed transcortical channels on the proximal articular surfaces of the tibia and femur^24^ and age-related non-traumatic pathology. Phalanx 1 of digit IV and proximal and middle caudal vertebrae of *G. mongoliensis* bear calcium deposits indicative of the calcium pyrophosphate deposition disease (CPPD; Fig. 2–4)^18^.

**Table 1:** Measurements (in cm) of ZPAL MgD-III/3 in comparison to the largest representatives of *Gobihadros mongoliensis* reported by Tsogtbaatar et al.^17^.

	Phalanx 1-II length	Phalanx 1-III length	Phalanx 1-IV length	Width of the caudal part of the proximal end	Tibia length	Proximal femur width	Femur length
MPC-D100/744				16.2	70.3	16.4*	72.8
MPC-D100/746				5.5	38.3	8.8	41.5
MPC-D100/751	6.5	7	5.3				
ZPAL MgD-III/3	9.2	11.5	9.3	19	82.4*	23.2	110*

Asterisks indicate lengths estimated from proportions due to the incompleteness of the specimens.

The abnormal area is distinguished from osteophytes and enthesophytes, because the former are limited to joint edges and the latter, because their position does not correspond to sites for attachment of ligaments or tendons^25^. The pathology is recognized as CPPD because of the classic presence of calcific concretions on joint surface^18,20^ (Fig. 4e). The radiodensity of the outgrowth is comparable to that of surrounding normal cortex. Most of its base is separated from the underlying surface of the bone by a radiolucent band. Underlying bone trabeculae are visible. Their structure and density appear normal, with no detectable disturbance in relation to the overlying pathology. The presence of calcium deposits indicates calcium pyrophosphate deposition disease (CPPD)^18^. In the absence of post-traumatic changes in the affected bones, we consider it as primary CPPD^18^, as opposed to representing a complication of osteoarthritis. Joint disease, often referred to as arthritis typically produces pain, stiffness, swelling and decreased range of motion^26^, but it is only its physical impact that can be recognized in the fossil record^27^. The diagnosis of CPPD is based on chondrocalcinosis (calcifications on joint surfaces) or pseudogout (presence of inflammation)^18^. CPPD is characterized by the accumulation of calcium pyrophosphate dihydrate crystals in the joint cartilage, probably caused by increased level of adenosine triphosphate (ATP)^28^. Risk factors for its development are ageing, osteoarthritis, trauma, metabolic diseases and genetic predisposition. CPPD is rare in human patients younger than 55 years^29^. In those cases occurrence is a complication of metabolic diseases such as hemochromatosis (iron storage disease), primary hyperparathyroidism, hypomagnesemia, hypothyroidism, ochronosis (homogentisic acid storage disease), or Wilson’s (copper storage disease)^28–30^. Secondary CPPD (as a complication of trauma-related osteoarthritis) was recognized only r ecently in the pes of a basal neornithischian^31^. Because the closed transcortical channels on the femoral and tibial articular surfaces (Fig. 5a), its body size (Table 1), and the histological sections (Fig. 5d,f) indicate that the *Gobihadros mongoliensis* individual was advanced in age^21^, we suggest that the CPPD (Fig. 5b–c,e) was primary, caused rather by ageing than metabolic disease. This is further supported by the absence of post-traumatic changes on the bones. The affected areas (tail and left pes) had restricted mobility, likely causing pain before the death of the animal. The afflicted *Gobihadros mongoliensis* reported herein appears to be the first non-avian dinosaur identified with primary CPPD, unrelated to osteoarthritis or trauma.

Ossification of nearly all cranial sutures is a frequently cited indicator of old age for representatives of various tetrapod groups^7^, However, the cranial sutures can either indeed progressively close, stay open, or even widen during ontogeny in recent archosaurs^32^. The sequence and timing of the fusion also differ between individuals^33^. Therefore, the utility of cranial suture fusion during ontogeny for ontogenetic age estimation is ambiguous. Similar to cranial sutures, fusion events in the post-cranium^6^ should also be taken with caution, because linear growth can still occur after co-ossification of the post-cranial bones (e.g., sternal ribs, epiphyseal ossifications, scapula with the coracoid)^2^.

Fusion of vertebral centra is found in sauropodomorphs^34–36^, theropods^37^, and reported in hadrosaurids^38^. Furthermore, it appears that among non-avian dinosaurs, fused contiguous vertebrae appear regardless of the ontogenetic age and are rather a result of spondyloarthropathy or diffuse idiopathic skeletal hyperostosis (DISH)^34–36^. Spondyloarthropathy may be caused by healing after trauma^38^ or mechanical stress and limited motion of the affected area^35^. DISH, on the other hand, is associated with the ossification of the vertebral longitudinal. It is not a form of arthritis and is considered rather a physiological phenomenon serving to protect from back pain than a disease^36,39^.

Relatively large body size is most used as a correlate of a senile age^6^. Accounting for the incompleteness of the fossil record leading to many extinct taxa being known from single or very few specimens^9,14^, “large size” is usually purely subjective. In some cases tracks reveal existence of individuals larger than those known from body fossils, but such individuals are rare^40^. The ichnological record gives limited information about the morphology, physiology, and ageing process of the trackmakers, on top of the usual uncertainty concerning the specific identification of the trackmaker. Furthermore, body size is subject to sexual dimorphism and significant variation within populations, being influenced by a number of intrinsic and extrinsic factors, such as the genetic makeup of the individuals and environmental conditions, including food availability and pathogen activity^41^. Additionally, growth in general ceases or significantly slows down around the time of sexual maturation, making this criterion useless for distinguishing adult and senile individuals.

The presence of EFS or OCL is commonly used in assessing the ontogenetic age of dinosaurs^9–12,42^. The utility of this feature is justified, because both the EFS and OCL constitute an evidence of slow tissue deposition and functional stoppage (or near stoppage) of growth. The study of a *Maiasaura peeblesorum* population revealed that even bones referred to subadults (based on their size) can already show the beginning of the EFS formation, so all adults should have the EFS in their bones^14,43^. This agrees with the observations on modern amniotes that the growth usually ceases or severely slows down around the moment of reaching the sexual maturity^44–46^ and the development of non-growth-related adult characteristics may be fully realized afterwards. However, bones of small circumference gain the EFS comparatively earlier than the thicker long bones^43^. Generally, the presence of EFS or OCL marks the slowdown or stoppage of the periosteal apposition of new bone layers, and thus slowdown or termination of the long bone radial growth (girth increase). Thus, it marks the termination of long bone thickening as the animal reaches terminal size. The rareness of EFS or OCL in sampled dinosaurs reveals how many of the sampled individuals did not reach the terminal size before their death, even though their skeletal anatomy frequently seems adult^21–23,47^. Most of the cortex thickness of weight bearing bones of almost all sampled eusauropods and large hadrosaurids is built of uninterrupted fibrolamellar complex^9,13,22,23,47–49^, which probably allowed them to attain large sizes^21,48^. This mostly records intensive bone deposition prior to somatic maturity. After deposition of the EFS or OCL subsequent life is not recorded histologically, aside from progressing secondary remodeling. Thus, the EFS or OCL mark the severe reduction of growth of the animal, which is connected to its skeletal maturity, but not necessarily advanced age. In addition to the termination of radial growth, cessation of growth connected to bone elongation can be observed in Archosauria on the proximal surfaces of the humerus, tibia, and femur. The transcortical channels perforating those surfaces provide blood supply for the articular cartilage responsible for bone elongation. When the channels close or disappear, longitudinal bone growth ceased and the terminal size of the animal is attained^50^. The transcortical channels ae observed in most of the non-avian dinosaurs (Sauropoda, Hadrosauridae, Iguanodontia, Hypsilophodontidae, Ceratopsia, Stegosauridae, Camptosauridae, and Theropoda) and their closure or loss is detected within many of the mentioned groups (Sauropoda, Hadrosauridae, Ceratopsia, and Theropoda), so it appears that the closing or loss of the transcortical channels seems to be a good indicator for skeletal maturity^24^.

The close temporal association between the cessation as measured by EFS of growth and sexual maturity in many animals is noteworthy in that context, and even in taxa with seemingly indeterminate growth, growth intensity significantly decreases after the sexual maturity^44–46^. This correlation appears to be plesiomorphic for reptiles and is frequent in mammals, although in numerous reptiles (particularly squamates, male crocodilians^51,52^) and mammals, the growth may continue well after the sexual maturity is reached. While in some species of mammals the disparity between the attainment of the sexual maturity and growth cessation may reach up to several years, in most cases it constitutes at most only several percent of average life expectancy. Usually post-sexual maturity growth amounts to only about 15 or less percent of terminal size of the animal^44,46,53^. in many cases. The growth data for extant animals is often cited in form of mass measurements. This may not accurately reflect linear, osteological growth (as recorded in the fossil record), since animals may continue to gain weight long after reaching their terminal linear sizes^46^. This is particularly pertinent since numerous dinosaur ontogeny studies cite elephants as sexually maturing long before reaching the terminal size, even though in fact they tend to achieve about 90% (males) to 95% (females) of their linear height at the moment of sexual maturity. In contrast to most amniotes, birds tend to exhibit an intensive growth spurt in their early life and reach sexual maturity significantly after reaching the terminal size^54^. The relationship between the sexual maturity and growth termination in dinosaurs is ambiguous. A study by Erickson et al.^55^ concluded that in maniraptorans the attainment of the sexual maturity occurred at most a few seasons earlier than that of the somatic maturity if not approximately at the same time. That interpretation is based on the observation that the brooding individuals approach the terminal sizes and in most cases that their bone cortices exhibit EFS. Conversely, the study by Lee and Werning^56^ suggested a much earlier sexual maturity in theropods and ornithopods, based on the presence of medullary bone as it forms a calcium source prior to egg production in sexually mature birds^57^. This interpretation may be in error, as medullary bone also identified in the mandibular symphysis of an azhdarchid pterosaur, despite the lack of hard eggshells in this group^58^. This suggests that medullary bone may be plesiomorphic for Ornithodira and not necessarily associated with reproduction, but rather subsequently adapted for that function within the bird lineage. As such, medullary bone may not be a reliable indicator of sexual maturity in non-avian dinosaurs. Moreover, at least some reports of medullary bone in dinosaurs may in fact represent post-fracture bony calluses^59^. O’Connor et al.^60^ suggested that early sexual maturity might have been plesiomorphic for birds, based on the presence of two closely spaced lines of arrested growth (LAGs) in the cortices of Early Cretaceous birds. This was interpreted as an indicator of the onset of sexual maturity. However, a study by Woodward et al.^61^ documents that similarly closely spaced LAGs occur in theropods at various stages of life and more than once in a single section, and that double LAGs can occur even in the middle and outer cortices of sexually mature xenarthrans^62^. Thus, this argument is rendered questionable. More compelling evidence is presented recently in form of a presumably gravid oviraptorosaur with associated eggs^63^ and a gravid enantiornithine^64^, both lacking OCL or EFS. This suggests that the specimens were reproductively active before reaching somatic maturity, but not how far removed are sexual and somatic maturity from each other temporarily or how much growth the specimens had still before them, nor whether the same growth strategy occurred in other dinosaur groups, such as large ornithopods. Woodward et al.^14^ hypothesized that sexual maturity occurred between the second and third year of life in *Maiasaura peeblesorum* (compared to skeletal maturity around the eight year of life), based on deceleration of growth rate, subtle increase of mortality and change of vascular orientation in the bone cortices. Such rapid sexual maturation seems unexpected, since the age of sexual maturity is well and positively correlated both with the body mass and expected adult lifespan at least in recent mammals and birds^54,65^. *M. peeblesorum*’s achievement of sexually maturity between the second and third year surpasses most reptiles, as well as large (and even many middle-sized) mammals, and approaches that of much (at least an order of magnitude) smaller and short-lived mammals and some birds^54^. Note that this estimate contrasts with that of Dunham et al.^66^, who suggested sexual maturity for *M. peeblesorum* at an age of at least five, and most likely 10 to 12 years. At this point the animal only achieves 36% of the asymptotic mass, while its linear size is already 80% of its asymptotic size^14^. Sander^9^ suggested sexual maturity in sauropods at 40–70% of the terminal size, based on the decrease of spacing between bone modulations visible on polished sections. Chinsamy-Turan^67^ reported that the thickness between zones can be variable and proposes correlation with other factors (e.g., growth conditions). Sander^9^ noted that this could represent an event during juvenile ontogeny and that the maturity could be reached closer to terminal size. There is controversy as to achievement of sexual maturity. Dunham et al.^66^ suggested that life histories with sexual maturity attained over the 20th year of life are unlikely to exist. This contrasts with the age of sexual maturity of bowhead whales at 25 years^68^ and some turtles at 45 years^69^. This line of reasoning is carried over to later works^22,23^, albeit with reservations, but Griebeler et al.^70^ assumed that at the age of sexual maturation the animal has achieved approximated reaching of 90% of its asymptotic mass. According to Kilborn et al.^44^, the ratio of the age of sexual maturity to the average lifespan in humans and laboratory mammals is less than 15%, and usually about 5%. Similar ratios can be inferred based on the much larger dataset gathered by Ricklefs^54^ for captive tetrapods: mean ratios are 7.68% (σ = 3.33%, N = 153) for mammals, 7.55% (σ = 4.07%, N = 74) for birds, 14.95% for reptiles (σ = 11.06%, N = 19), and 16.44% for amphibians (σ = 9.3%, N = 5) (note that the maximal lifespans of the animals in this datasets may be underestimated due to limited sample sizes of captive exotic species but also that these ratios would likely be higher in the wild due to the increased mortality and lesser probability of attaining old ages). Nonetheless, still a large portion of animals’ life, including most or all their adult life, is not associated with growth. In summary, cessation of longitudinal and perhaps radial growth appear to be considered a prerequisite for the identification of a senile animal.

Multiple generations of secondary osteons were previously used as an indicator of advanced age^13,22,23^. This phenomenon is incorporated in three staging schemes—the Histologic Ontogenetic Stages by Klein and Sander^22^, the modified four-stage scheme for *Stegosaurus* spp. by Hayashi et al.^11^, and the Remodeling Stages by Mitchell et al.^13^. Generally, after the deposition of the EFS, the bone remodeling continues reaching the external most cortex. The expansion of secondary remodeling is connected to ageing and results from mechanical stress or strain affecting bones during life^71^. Secondary remodeling density was examined in sauropods^13,22,23^, stegosaurs^11^, and ankylosaurs^72^. The largest sauropods reveal almost completely remodeled primary bone of their humeri and femora^13^. This is a potential indicator of the advanced age, although expansion of secondary remodeling can differ between the bones within a given individual and along the bone, depending on the mechanical stress. The main expansion of remodeling occurs after cessation of growth, as expressed by the closure or loss of transcortical channels on the proximal articular surfaces of long bones and formation of the EFS, when no new primary bone tissue is deposited either longitudinally or radially. Because the bone was no longer growing, secondary osteons could reach the external-most cortex and new generations of the osteons were created with time, marking the senile age of the animal. The remodeling is also extensive in the largest sampled specimens of *Stegosaurus* spp. (called “old” by Hayashi et al.^11^; these likely represent adults as they have the EFS and sometimes more than one generation of secondary osteons), but relatively lesser compared to the largest sauropods^11^. On the contrary, remodeling appeared much earlier in ankylosaur ontogeny and is overall much stronger^72^.

Both the Histologic Ontogenetic Stages and Remodeling Stages are most informative in comparative context, to establish relative age of specimens belonging to the same or closely related species. Unfortunately, remodeling-based methods have several limitations possibly making differentiation between, e.g., older adults and senile individuals difficult. Firstly, it is not possible to predict the maximal possible degree of remodeling for any given species. The most remodeled sampled specimen of a given taxon is assumed to be the oldest and likely senile, but this makes the method relative and prone to errors due to under sampling. Secondly, remodeling may potentially lose resolution in the latest stages of ontogeny, as new generations of osteons are developed in an already remodeled tissue, increasingly overwriting the previous record. This may be particularly problematic for species with the longest lifespans, in which multiple generations of osteons may develop over time, and in species in which the osteons are relatively larger compared to the cortex thickness (e.g., hadrosaurs versus sauropods^21,23^). In our samples of *Gobihadros mongoliensis* we observe multiple cases of new osteons developing centered on the older ones, which also may substantially influence the observed number of generations, especially at more advanced stages of remodeling (Fig. 5). Thirdly, viability of stage comparisons between different distantly related species is yet to be evaluated. Since the physiology of bone deposition and remodeling in dinosaurs is still being studied and may differ between various taxa, there are several unanswered questions:

Is the rate of remodeling in any way dependent on the average life expectancy? I.e., does the same number of osteon generations in two unrelated dinosaur taxa indicate the same amount of time since reaching the somatic maturity, regardless of their total lifespan (absolute age)? Or does the rate of remodeling indicate the same life stage relative to the maximal biologically viable ontogenetic age, regardless of the actual year count since the somatic maturity (relative age)? While resemblances between sauropods and ornithopods were presented before^21^, studies on thyreophorans^11,72^ reveal that at least for some clades modifications of the staging schemes are necessary and that the stages should be calibrated for each group individually, based on large samples.

The herein presented individual of *Gobihadros mongoliensis* manifests the terminal size of the species, determined by the presence of the EFS in its femur and closed transcortical articular surface channels. Its senescence is supported by extensive remodeling reaching the external surface of the cortex and age-related pathology (Fig. 5). It is the first non-avian dinosaur identified with primary CPPD. Thus, we would like to propose a definition for a senile non-avian dinosaur, as follows: an individual which achieved the terminal size as revealed by the presence of the EFS and closure of transcortical channels, secondary remodeled weight-bearing bones (femora and humeri, if quadrupedal) and non-traumatic, non-contagious bone pathologies correlated with advanced age (e.g., primary CPPD). The proposed definition is important for revision of the non-avian dinosaurs previously suggested as senile, but may just represent individuals which just terminated their growth. The discern between morphologically adult and senile specimens is important for identifying additional changes in the skeleton during the ca. 90% of lifespan after the termination of growth. Moreover, the definition of a senile individual gives an opportunity to reassess specimens previously identified as senile based on features which can appear earlier in ontogeny (e.g., fused vertebrae). Thus, some of the features now regarded as senile may actually represent normal, adult skeleton changes, characteristic for the species, which appear after the termination of growth, when the animal was in the prime of its life. Although the pathologies are not reported in many studied individuals, they constitute a line of evidence independent from histology. Future studies are encouraged to note even mild abnormal morphologies as additional guides to estimation of the ontogenetic stages of dinosaurs.

## Material and methods

The specimen ZPAL MgD-III/3 was collected in Khongil Tsav, Mongolia; it was found in the sands and mudstones of the Baynshire Formation in 1963^73^. According to field notes, the bones were found in an association and likely belong to a single individual. The fossils are deposited in the collection of the Institute of Paleobiology, Polish Academy of Sciences in Warsaw. The Baynshire Formation is estimated to be late Cenomanian to Santonian in age, positioned between the Cenomanian Sainshand Formation and the middle Campanian Djadokhta Formation^74^. Multiple hadrosauroid specimens considered as subadults and adults from this formation were described under the name *Gobihadros mongoliensis* by Tsogtbaatar et al.^17^. The individual ZPAL MgD-III/3 consists of fragmentary left metacarpals, proximal fragments of the right ilium and ischium, proximal and distal parts of the right femur, proximal right tibia, fragmentary left pes, nine caudal vertebrae, and several smaller bone fragments. Due to the incompleteness of the femur and tibia of ZPAL MgD-III/3, the total lengths of these bones can only be estimated. Nevertheless, size comparisons of the preserved proximal parts indicate that the individual was larger than MPC-D100/744 (Mongolian Paleontological Center, Ulaanbaatar, Mongolia), the individual of *G. mongoliensis* with the largest femur and tibia reported so far^17^ (Table 1). The estimations of the femur and tibia lengths were based on the complete femur of the individual MPC-D100/746 figured in Tsogtbaatar et al.^17^. The pes of the latter is unknown, so the lengths of the proximal phalanges of the digits II, III, and IV of ZPAL MgD-III/3 were compared to MPC-D100/751, the largest pes of *G. mongoliensis* reported so far^17^ (Table 1). The tibia and femur of the *G. mongoliensis* individual ZPAL MgD-III/3 show signs of a cessation of growth: closely spaced lines of arrested growth^21^ (observable in the femur, likely obscured by the extensive remodelling in the tibia) and closed transcortical channels on articular surfaces^24^. When those channels close, access to nutrient for longitudinal bone growth is lost and elongation ceases. Thus, it appears that the individual shows the terminal size of *Gobihadros mongoliensis.* See Supplementary Table S1 for detailed measurements of the specimens.

The animal exhibits pathologies on the proximal and middle caudal vertebra and pes. The pathological proximal caudal vertebra and phalange were scanned using the Nikon/Metris XT H 225 ST computed tomographic (CT) unit housed at the Military University of Technology, Warsaw, Poland at 220 kV voltage and 81 μA current intensity. The CT images were captured using 2000 × 2000 pxs scintillator (1000 projections) Obtained slices were processed using the Volume Graphics® MyVGL viewer app and GOM Inspect. Remaining bones were scanned using the Shining 3D EinScan Pro 2X 3D scanner fixed on a tripod with EinScan Pro 2X Color Pack camera (texture scan), motorized Ein-Turntable (alignment based on features), and EXScan Pro 3.4.0.4 software. Meshing was done using the Watertight Model and Medium Detail pre-sets. The snapshots of the 3D models shown in the figures were captured in MeshLab 2020.07^75^ in orthographic view and with the Radiance Scaling (Lambertian)^76^. The obtained models are available for download as Supplementary Models 1-28 (see Table S2 for the full list and anatomical identifications) in .PLY format, opened by the built-in 3D Viewer or Paint 3D apps in Windows 10, or MeshLab free software (https://www.meshlab.net/).

The middle caudal vertebra was thin sectioned in the Institute of Paleobiology, Polish Academy of Sciences. Large-scale photographs of thin sections were obtained using a Nikon Eclipse LV100 POL polarizing microscope with a DS-Fil camera in transmitted normal and polarized light, including a gypsum wedge. The pictures were combined together in NIS-Elements 4.20.01 64 bit microscope imaging software.

## Supplementary Information

Supplementary Model 1.

Supplementary ﻿Model 2.

Supplementary ﻿Model 3.

Supplementary ﻿Model 4.

Supplementary ﻿Model 5.

Supplementary ﻿Model 6.

Supplementary ﻿Model 7.

Supplementary ﻿Model 8.

Supplementary ﻿Model 9.

Supplementary ﻿Model 10.

Supplementary ﻿Model 11.

Supplementary ﻿Model 12.

Supplementary ﻿Model 13.

Supplementary ﻿Model 14.

Supplementary Model 15.

Supp lementary ﻿Model 16.

Suppl e mentary ﻿Model 17.

Supplementary ﻿Model 18.

Supplementary ﻿Model 19.

Supplementary ﻿Model 20.

Supplementary ﻿Model 21.

Supplementary ﻿Model 22.

Supplementary ﻿Model 23.

Supplementary ﻿Model 24.

Supplementary ﻿Model 25.

Supplementary ﻿Model 26.

Supplementary ﻿Model 27.

Supplementary ﻿Model 28.

Supplementary Information.
