# Supplementary material for: Dinosaur senescence: a hadrosauroid with age-related diseases brings a new perspective of “old” dinosaurs
Source: Sci Rep. 2021 Jun 11;11:11947. doi: 10.1038/s41598-021-91366-1 (PMC8196189; doi:10.1038/s41598-021-91366-1)
Supplement: Supplementary file 29 — Supplementary Information. [file 41598_2021_91366_MOESM29_ESM.pdf]

Supplementary Materials for

Dinosaur senescence: a hadrosauroid with age-related diseases brings a new perspective of "old" dinosaurs

Justyna Słowiak, Tomasz Szczygielski*, Bruce M. Rothschild, and Dawid Surmik

*Corresponding author. E-mail: t.szczygielski@twarda.pan.pl

Detailed descriptions of *Gobihadros mongoliensis* ZPAL MgD-III/3 bones.

All specimens are available for download as Supplementary Models. See Table S2 for the list of files and their identifications.

Metacarpals. The proximal end of the metacarpal III is mediolaterally flattened, but the metacarpal becomes oval in the cross section in the middle part. In the proximal view the articular surface is trapezoid, and the cranial margin is narrower mediolaterally than the caudal. The proximal end of the metacarpal IV is turned slightly cranially, and its surfaces are flat cranially, caudally, laterally, and medially, where it articulated to the metacarpal III. The lateral surface is rounded. The articular surface is heavily damaged. As preserved, its proximal outline is subtriangular, but the shape of the bone just below the damaged area suggests a trapezoid contour, as in the metacarpal III, although its acutest angle points laterally. Below, the lateral margin in cranial and caudal view is gently concave.

Pelvis. A small part of probable right ilium above the ischial peduncle is preserved. The lateral surface of the element is concave, and the caudal margin is curved medially. The preserved proximal part of the right pubis is weakly preserved and lacks informative characters. The caudal part of the iliac peduncle of the right ischium is preserved. The preserved proximal part is robust and strongly curved caudally, similar as in other individuals of *Gobihadros mongoliensis*[1].

Femur. The proximal and distal parts of the right femur are preserved. In proximal view, the articular surface of the proximal end is δ-shaped with the cranial concavity deeper than the caudal. The dorsal surface of the medially projected rounded head is rugose, suggesting the presence of a cartilaginous cap. The greater trochanter is mostly complete and compressed

lateromedially. It projects cranially more than caudally and its dorsal margin forms an arch. The greater trochanter is comparable to the femoral head in its craniocaudal width, similar as in basal Hadrosauridea. The lesser trochanter is not preserved. The saddle-like dorsal margin of the femur is consistent with the shape seen in other representatives of Hadrosauroidea. The robust and only slightly protruding medially femoral head and greater trochanter are comparable in craniocaudal length to the femoral head and fit the shape of the proximal femur of other specimens of *Gobihadros mongoliensis*[1]. The distal end is severely damaged, with most natural surfaces destroyed; only the medial condyle is preserved.

The distal end is severely damaged, with most natural surfaces destroyed, but seemingly only the medial condyle is preserved. The medial and caudal parts of the condyle are preserved best, with other parts damaged, but as is, the condyle is comma-shaped (caudal end turned laterally) and comparable in length to the greater trochanter of the proximal head. The intercondylar flexor groove was apparently deep. The medial surface of the medial condyle is flat with proximodistal striation.

Tibia. Only the proximal end of the right tibia is preserved. In proximal view, the tibia is lateromedially flattened and extended craniocaudally. The whole proximal surface is rugose, suggesting the presence of a cartilaginous cap. Transcortical channels are not detected. The caudomedial condyle is rounded in proximal view and its most proximal part is extending caudomedially. More cranially, on the medial side of the proximal tibia, the base of the lateral condyle is preserved. It is separated from the caudomedial condyle by a narrow and deep cleft. The lateral condyle is mostly broken, but its base indicates that it was narrow craniocaudally and projected caudomedially. The lateral surface of the proximal end of the tibia is relatively straight, with minor concavity at the base of the cnemial crest (ambiguous whether natural or

caused by crushing). Overall, the presence of a robust caudomedial condyle falls into the proximal tibia morphology of the *Gobihadros mongoliensis* specimens[1].

Foot. Left foot elements are preserved, including parts of distal metatarsals and phalanx 1 of digits II, III, and IV. Fragmentary remains of metatarsals consist of partial distal articular ends of the metatarsal II, III, and IV. The metatarsals are too fragmentary to perform meaningful comparisons. The general shape of the preserved elements of the metatarsals is similar to that of other hadrosaurids.

Only the medial half of phalanx 1 of the left digit II is preserved. Judging from the preserved part, the proximal articular surface is roughly rectangular in proximal view, nearly flat, with a slight caudal projection near the mediodorsal edge. The dorsal, medial, and especially the ventral surfaces are slightly concave. The distal articular surface is convex vertically. The ventromedial margin of the distal articular surface is expanded medially. The nearly symmetric phalanx 1 of left digit III is the largest. The proximal articular surface is not preserved. The lateral and medial surfaces are almost straight. Both the dorsal and the ventral surfaces are concave, the ventral more so than the dorsal. Convex in mediolateral and straight in dorsoventral aspect, the distal articular surface continues onto the distal dorsal surface. In the distal view, the lateral and medial margins of this surface gently converge dorsally. Phalanx 1 of the left digit IV is asymmetric; the lateral margin is longer than the medial. The proximal articular surface is flat and roughly trapezoid to subtriangular, with an especially pronounced medial margin. The dorsal surface of the phalanx is more concave than the lateral and ventral, while the medial surface is flat. The distal articular surface is slightly saddle-shaped, convex in mediolateral aspect. Its medial and ventral margins are straight, while the dorsolateral margin is continuous and gently bowed. The lateroventral part of the distal articular surface is not

preserved. There is a surface calcific deposit on the middle aspect of the dorsolateral edge of the distal articular surface, diagnostic of calcium pyrophosphate deposition disease. The general shape of the first phalanges of the digits II, III, and IV fall into the spectrum of shapes known in other Hadrosauroidea.

Caudal vertebrae. Parts of nine caudal vertebrae are preserved. Three of them represent the proximal part of the tail; four, middle; and the remaining two, distal. The centra of the vertebrae from the proximal part of the tail are dorsoventrally higher than craniocaudally long. The height of middle caudal vertebrae is similar to their length and the distal caudal vertebrae are longer than high.

Proximal caudal vertebrae. The centrum of the most proximal of the preserved caudal vertebrae has a circular and slightly concave cranial surface. The caudal surface is poorly preserved, but both surfaces seem to be roughly parallel. The caudoventrolateral right margin of the centrum bears a large ovoid articular surface for the chevron (the left one is not preserved). There is no sign of such an articular surface at the cranial end, which may indicate that this vertebra is from the very base of the tail, right behind the sacrum. The centrum has slightly concave lateral surfaces and the ventral surface is strongly concave. The spinous process is not preserved. Only the base of the neural arch remains, suggesting that it was located closer to the cranial edge of the vertebra. The base of the transverse process is dorsoventrally flattened and directed laterally, slightly downward and concave cranially, resulting in an arrowhead-shaped cross-section. The distal portions of the processes are not preserved. Only the dorsolateral right portion of another, slightly more distal caudal vertebra centrum is preserved. The centrum seems to be sub-rectangular, higher than wide. Both the cranial and the caudal surfaces of the centrum are damaged. The preserved parts are slightly concave and the

cranioventral margin of the centrum has a rounded articular surface for the chevron, which is slightly smaller than in the anterior-most preserved caudal vertebra. The lateral surface of the centrum is straight. The last preserved vertebra of the proximal part of the tail is poorly preserved. The base of its spinous process is closer to the cranial edge of the dorsal surface of the vertebra. Only the base of the right transverse process is preserved. It is directed laterally and is triangular in cross section. The ventral surface of the transverse process is flat, while the dorsal corner is directed craniodorsally.

Middle caudal vertebrae. Only half of an anterior middle caudal vertebra is preserved. The position of the neural arch (supposedly closer to the anterior edge) suggests that the right half is represented. The cranial and caudal surfaces of the centrum are roughly straight. The cranioventral margin of the centrum bears an articular surface for the chevron, which is similar in size to the analogous surface on the posterior proximal caudal vertebra. The chevron facet is covered by a calcium deposit, indicating CPPD. The lateral margin is more concave than in the preceding vertebrae and there is no sign of transverse processes. The preserved base of the neural spine is projects straight dorsally. The right side of another, smaller middle caudal vertebra is preserved. The cranial and caudal surfaces of its centrum are slightly concave and an articular surface for the chevron, smaller than on the previous vertebrae, is present on the caudoventral margin, which is. A damaged remnant of a possible articular surface for the chevron is also present cranioventrally, but its incompleteness prevents definitive identification. The ventral surface is strongly concave, but the neither the lateral surfaces nor the spinous and transverse processes are preserved. The right surface of the third most distally preserved middle caudal vertebral centrum has a strongly concave ventral surface, but the lateral surface is only slightly concave. No other features are preserved. The ventral concavity of the fourth, most distally preserved middle caudal vertebra preserved is less concave ventrally than the previous

middle caudal vertebrae. It exhibits curvature similar to that of the right lateral surface. Only small parts of natural cranial and caudal surfaces are preserved, which exhibit gentle concavities.

Distal caudal vertebrae. The two preserved distal caudal vertebrae are incomplete, represented mainly by the middle parts of the centra. In contrast to the described above, they are longer craniocaudally than high dorsoventrally.

The morphology of the proximal caudal centra (i.e., short craniocaudally, wide mediolaterally with larger distal than proximal surfaces for chevron articulation), middle caudal centra (i.e., dorsoventral height similar to the craniocaudal length, prominent proximal and distal articular surfaces for the chevron and lack of transverse processes), and distal caudal centra (i.e., cylindrical centra twice as long craniocaudally than high dorsoventrally) is the same as the caudal vertebra of the *Gobihadros mongoliensis* individuals already described[1].

Other elements. Four fragmentary bones were also preserved. (I) An apical fragment of a transverse processes of a vertebra with flat ventral surface and convex dorsal surface. The surface of the apex is rugose and a bit swollen ventrally, suggesting the presence of a cartilaginous finish. (II) A larger flat bone with longitudinal striation, which may represent a rib, or (if it was symmetric in life) a neural spine. (III) A small fragment of a caudal zygapophysis is preserved, but its exact position in the column is not determinable. Moreover, (IV) a middle part of a chevron is preserved. The bases of the two processes forming the haemal canal suggest that the processes were close to each other, so the haemal canal was narrow. Underneath, the chevron is oval in cross section and the cranial surface is parallel to the caudal surface. The cranial surface is gently concave and the caudal surface exhibits stronger concavity

under the haemal canal. Below that, narrow ridges run along the chevron both on the cranial (less pronounced) and the caudal (more pronounced) surface. All these features imply that the chevron is from a proximal part of the tail. The chevron does not display notable differences with other hadrosaurs.

References

1. Tsogtbaatar, K., Weishampel, D. B., Evans, D. C. & Watabe, M. *A new hadrosauroid (Dinosauria: Ornithopoda) from the Late Cretaceous Baynshire Formation of the Gobi Desert (Mongolia)*. *PLoS ONE* vol. 14 (2019).

Histological sections

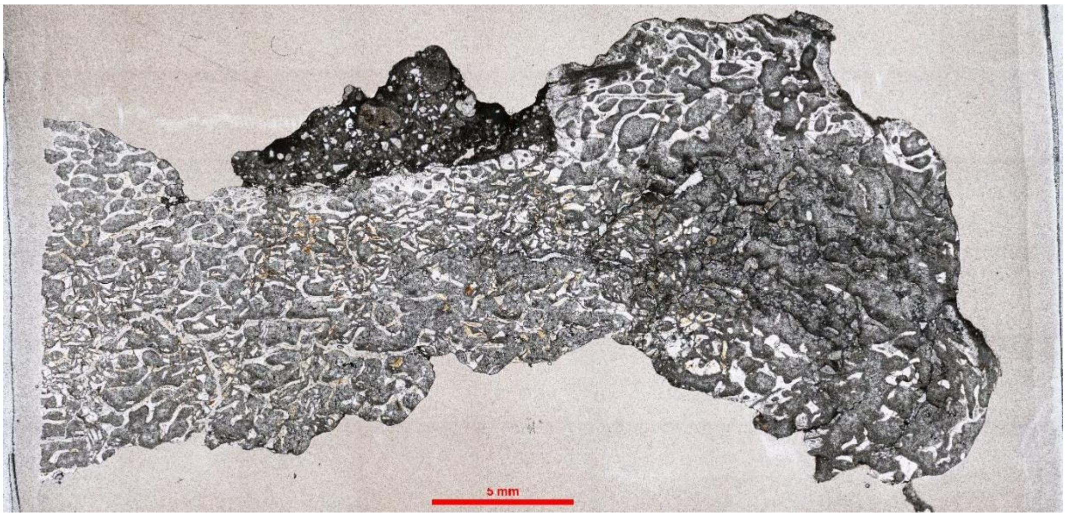

Figure S1. ZPAL MgD-III/3, thin section of the middle caudal vertebra under normal light.

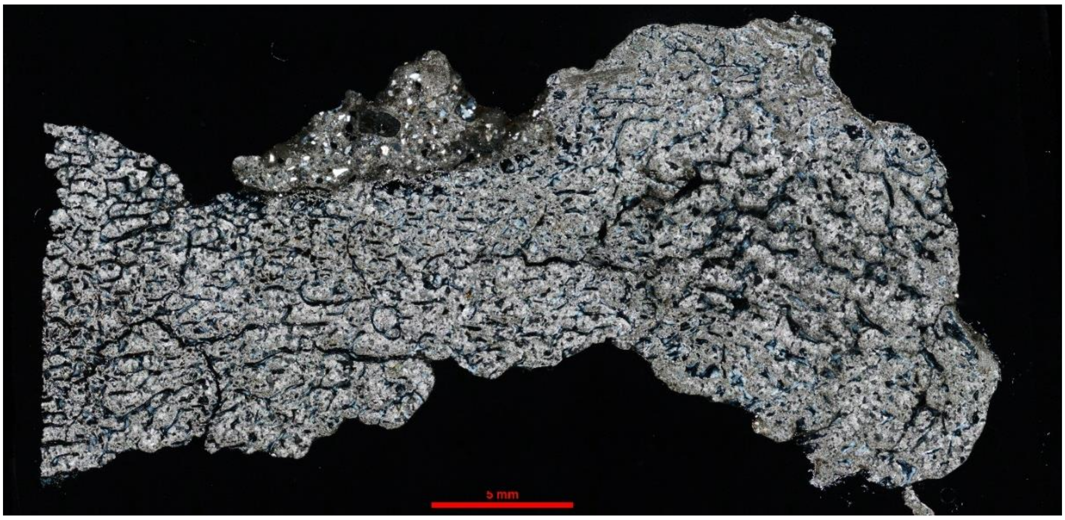

Figure S2. ZPAL MgD-III/3, thin section of the middle caudal vertebra under polarized light.

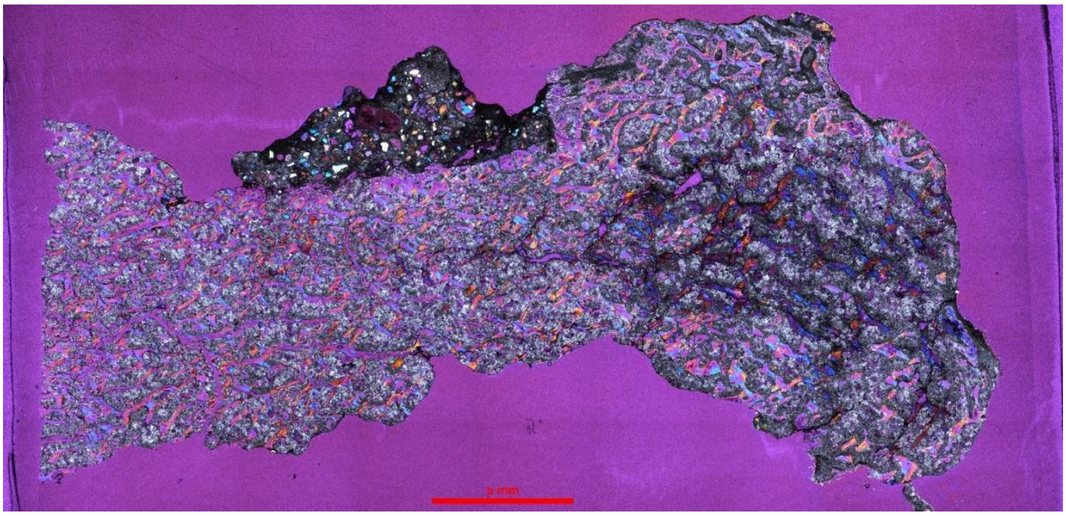

Figure S3. ZPAL MgD-III/3, thin section of the middle caudal vertebra under polarized light with gypsum wedge.

Table S1. Measurements of *Gobihadros mongoliensis* ZPAL MgD-III/3, in cm. * indicates measurements taken as preserved of damaged or broken bones.

**Metacarpals**				
	Length		Lateromedial width of the proximal end	Craniocaudal width of the proximal end
Metacarpal III	68*		28	35
Metacarpal IV	109*		29*	34*
**Long bones of the hindlimb**				
	Craniocaudal width of the proximal end		Lateromedial width of the proximal end	Craniocaudal width of the distal end
Femur	82 (midlength)<br>123 (femoral head)<br>142 (greater trochanter)		232	155*
Tibia	102* (midlength)<br>87 (caudomedial condyle)<br>73* (cnemial crest)		253*	-
**Foot**				
	Craniocaudal length		Lateromedial width in mid-length	
Phalanx 1 of digit II	92.31		26*	
Phalanx 1 of digit III	115.97		58	
Phalanx 1 of digit IV	93.2		45	
**Caudal vertebra**				
	Dorsoventral height of the centrum	Mediolateral width of the centrum	Dorsal craniocaudal length	Lateral craniocaudal length
Proximal caudal vertebrae	115	115	68	66
	101	80*	69	66
	94*	83*	65	59*
Middle caudal vertebrae	83	62*	77	71
	75	34*	68	66
	73*	25*	64	65
	43*	27*	53*	6
Distal caudal vertebrae	33*	37	47*	44*
	35*	32	49*	54*

Table S2. 3D models of *Gobihadros mongoliensis* ZPAL MgD-III/3 bones.

Label	Anatomical identification
Supplementary Model 1	Distal caudal vertebra 1
Supplementary Model 2	Distal caudal vertebra 2
Supplementary Model 3	Middle caudal vertebra 1
Supplementary Model 4	Middle caudal vertebra 2
Supplementary Model 5	Middle caudal vertebra 3
Supplementary Model 6	Middle caudal vertebra 4
Supplementary Model 7	Proximal caudal vertebra 1
Supplementary Model 8	Proximal caudal vertebra 2
Supplementary Model 9	Proximal caudal vertebra 3
Supplementary Model 10	Chevron
Supplementary Model 11	Femur, right, distal part
Supplementary Model 12	Femur, right, proximal part
Supplementary Model 13	Ilium, right
Supplementary Model 14	Ischium, right
Supplementary Model 15	Metacarpal III
Supplementary Model 16	Metacarapal IV
Supplementary Model 17	Metatarsal 1, left, distal part
Supplementary Model 18	Metatarsal 2, left, distal part
Supplementary Model 19	Metatarsal 3, left, distal part
Supplementary Model 20	Neural spine or rib fragment
Supplementary Model 21	Phalanx 1 II, left
Supplementary Model 22	Phalanx 1 III, left
Supplementary Model 23	Phalanx 1 IV, left
Supplementary Model 24	Tibia, right, proximal part
Supplementary Model 25	Transverse process
Supplementary Model 26	Unidentified bone fragment 1
Supplementary Model 27	Unidentified bone fragment 2
Supplementary Model 28	Zygapophysis
